# Silico-Tuberculosis Mimicking Malignancy

**DOI:** 10.7759/cureus.65411

**Published:** 2024-07-26

**Authors:** Ragavi Elango, Ashwin Kailash, Ghanshyam Verma, Prasana Rudhramoorty

**Affiliations:** 1 Respiratory Medicine, Sree Balaji Medical College and Hospital, Chennai, IND

**Keywords:** acid-fast bacilli, occupational hazards, bronchoscopy, tuberculosis, silicosis

## Abstract

Silico-tuberculosis is the combination of silicosis and tuberculosis (TB). Symptoms of TB such as dyspnea, cough, and hemoptysis may precede the diagnosis of an endo-bronchial mass lesion on chest imaging. Among workers who were exposed to silica, TB was more common, and experiments indicated that silica inhibits alveolar macrophage activity and severe exposure induces apoptosis. Endobronchial neoplasms, which are tumors primarily affecting the bronchial lumen, are uncommon and may show up in a wide variety of different ways pathologically. Cough, chest discomfort, wheezing, hemoptysis, recurrent pneumonia, and weight loss are common complaints from people with endobronchial tumors. The requirement for additional bronchoscopy and imaging examination is raised when symptoms such as hemoptysis and obstructive pneumonia are present. Endobronchial silicosis lesions are uncommon and develop as a result of broncho lithiasis, the endoluminal erosion of peri bronchial adenopathy, or local bronchial wall silica-induced fibrosis. Endobronchial TB can present in a varied manner, diagnosis is often challenging as there is no specific radiological feature, and sputum acid-fast bacilli several times come out negative. However, a bronchoscopy with or without biopsy is a useful investigation in these cases. The following case is a rare manifestation of endobronchial TB as it mimicked malignancy, describing the difficulties in diagnosis and treating a patient who had both silicosis and endobronchial TB.

## Introduction

Silica dust is the leading cause of silicosis, a chronic lung illness, and poses significant occupational health risks worldwide [1]. Crystalline silica dust exposure is linked to silicosis, silica dust-associated tuberculosis (TB), and lung cancer [2]. Individuals with silicosis have a 2.8 to 39 times higher risk of contracting pulmonary TB. Workers with the highest dust exposure are three times more likely to develop both malignancy and TB compared to those with lower exposures [3]. The risk of these diseases correlates with the severity of silicosis and exposure intensity [4].

Silico-TB is the combination of silicosis and TB. Symptoms like dyspnea, cough, and hemoptysis may precede the diagnosis of an endobronchial mass on chest imaging. TB is more common among workers exposed to silica, as silica inhibits alveolar macrophage activity and severe exposure induces apoptosis [5]. Endobronchial tumors, affecting the bronchial lumen, are rare and can present with cough, chest discomfort, wheezing, hemoptysis, recurrent pneumonia, and weight loss. Hemoptysis and obstructive pneumonia raise the need for further bronchoscopy and imaging. Endobronchial silicosis lesions, which are rare, develop due to broncholithiasis, endoluminal erosion of peribronchial adenopathy, or local bronchial wall silica-induced fibrosis [5]. The present case is notable for its resemblance to malignancy and highlights the co-existence of silicosis and endobronchial TB, as well as the diagnostic and treatment challenges encountered.

## Case presentation

A 40-year-old male, employed as a stone cutter for the past 20 years, presented to our emergency department with significant respiratory symptoms. His medical history includes 15 years of smoking with a smoking index of 400 and 20 years of alcohol use. He reported a persistent cough with expectoration and intermittent breathlessness persisting for the last two years. Recently, he has experienced hemoptysis over the past four days, accompanied by notable constitutional symptoms such as significant weight loss, poor appetite, and night sweats. His occupational exposure to silica dust and his smoking and alcohol history raise concerns for severe pulmonary pathology. 

Upon further inquiry, he disclosed a history of recurrent respiratory infections and night sweats. His occupational exposure to silica dust and his smoking history place him at high risk for pulmonary diseases. A thorough clinical evaluation and diagnostic workup, including chest imaging and bronchoscopy, are warranted to assess for potential silicosis and TB or other pulmonary pathology.

On examination, the patient was poorly built, and grade II clubbing and nicotine stains in the buccal mucosa and the palms were noted. On auscultation, bilateral air entry presented with bilateral normal vesicular breath sounds along with added sounds: bilateral suprascapular, interscapular, and intrascapular fine inspiratory crackles and bilateral wheeze with increased intensity in the left suprascapular, supraclavicular, and infraclavicular ones. All routine blood investigations were within normal limits.

**Figure 1:**
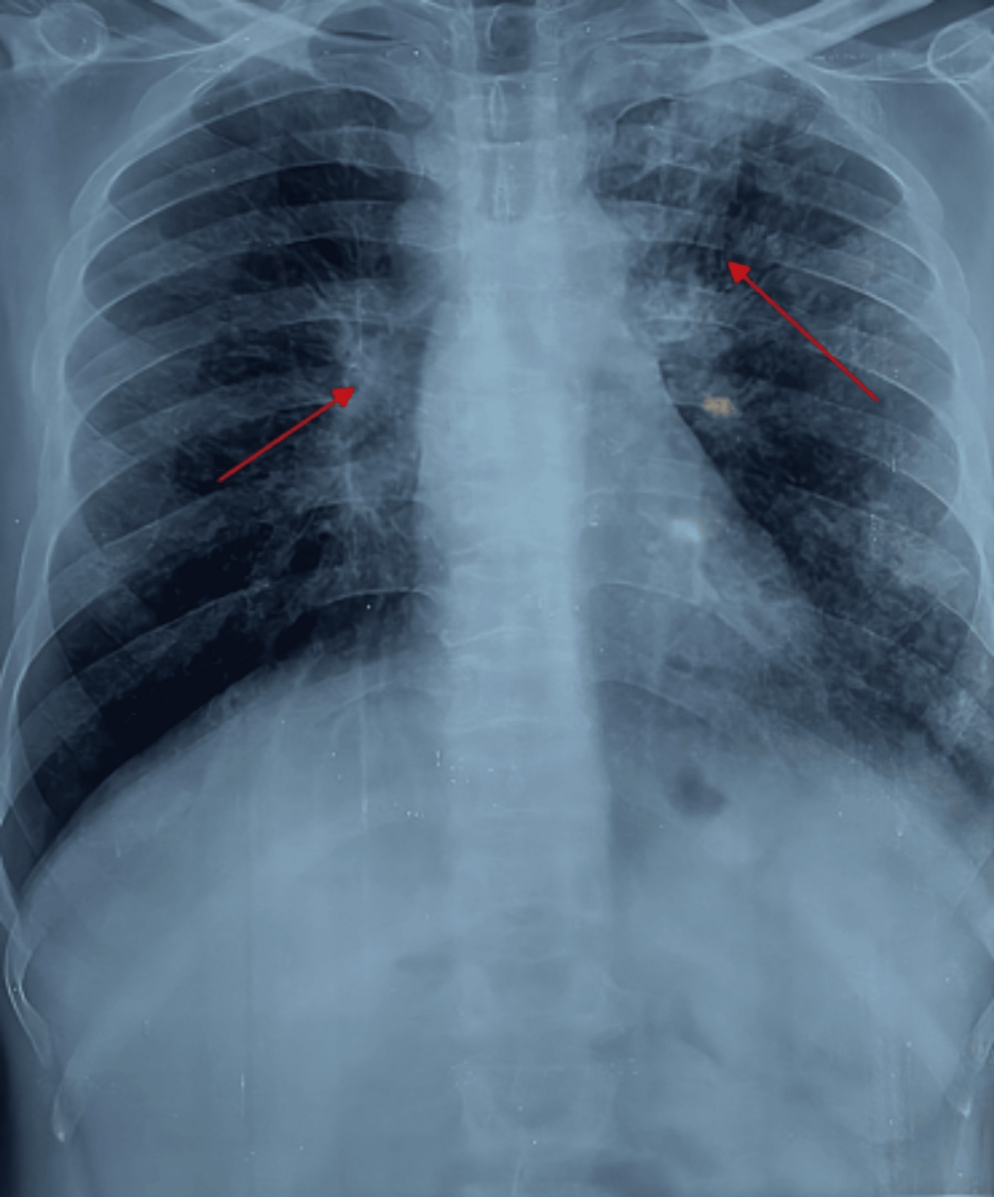
Chest X-ray-postero-anterior (PA) view showing bilateral diffuse nodular opacities

The chest radiograph (CXR-PA) shows bilateral lung fields exhibiting diffuse, small, rounded opacities predominantly distributed in the upper and middle zones, consistent with nodular infiltrates. There is evidence of progressive massive fibrosis, particularly in the upper lobes, characterized by coalescence of nodules and associated volume loss. The hilar regions appear prominently enlarged, potentially indicating lymphadenopathy. Fibrotic changes are noted, manifesting as increased linear opacities, especially in the upper lung zones. Subtle pleural thickening is observed, most notably at the lung apices. While the cardiac silhouette and mediastinum appear within normal limits, they are partially obscured by the overlying pulmonary abnormalities. The overall pattern of diffuse nodularity, upper lobe predominance, hilar enlargement, and fibrotic changes is classical for silicosis with superimposed TB. However, it is important to note that while these radiographic findings are highly characteristic, definitive diagnosis would require correlation with clinical history, occupational exposure data, and potentially additional diagnostic studies (Figure 1).

**Figure 2:**
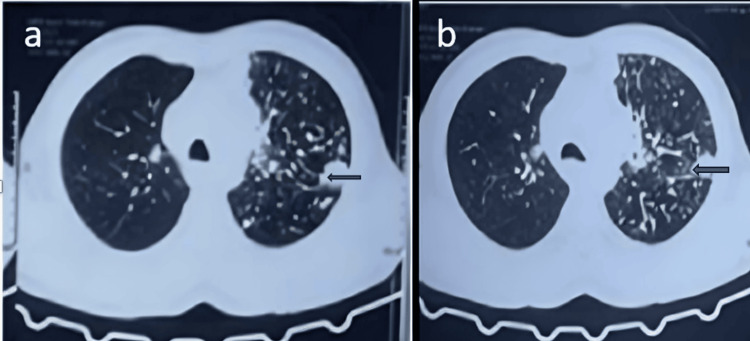
(a and b) High-resolution computed tomography (HRCT) thorax showing multiple nodular opacities in bilateral upper and lower lobes

High-resolution computed tomography (HRCT) of the thorax reveals significant abnormalities consistent with silicosis complicated by tuberculosis (silicosis-TB). Figure 2 shows diffuse involvement of both lung fields, with the left lung demonstrating more severe changes. Multiple small to medium-sized nodules are scattered throughout both lungs in a random distribution, with a tendency to coalesce, particularly in the upper and mid zones of the left lung. There is evidence of interstitial fibrosis, manifesting as linear opacities, with more pronounced fibrotic changes and architectural distortion in the left upper lobe. A potential cavity or area of consolidation is noted in the left upper lobe, which could indicate active TB. The upper lobe predominance of these findings is characteristic of both silicosis and TB. Volume loss in the left upper lobe is suggested by a slight elevation of the left hilum. While the right lung shows less severe involvement, it still demonstrates a nodular pattern consistent with silicosis. The progression shown in Figure 2A to Figure 2B indicates a worsening of the condition, with more pronounced fibrosis and potential cavity formation in the left upper lobe. These findings collectively support the diagnosis of silicosis-TB, showcasing the typical radiological features of this combined condition (Figures 2A, 2B).

**Figure 3:**
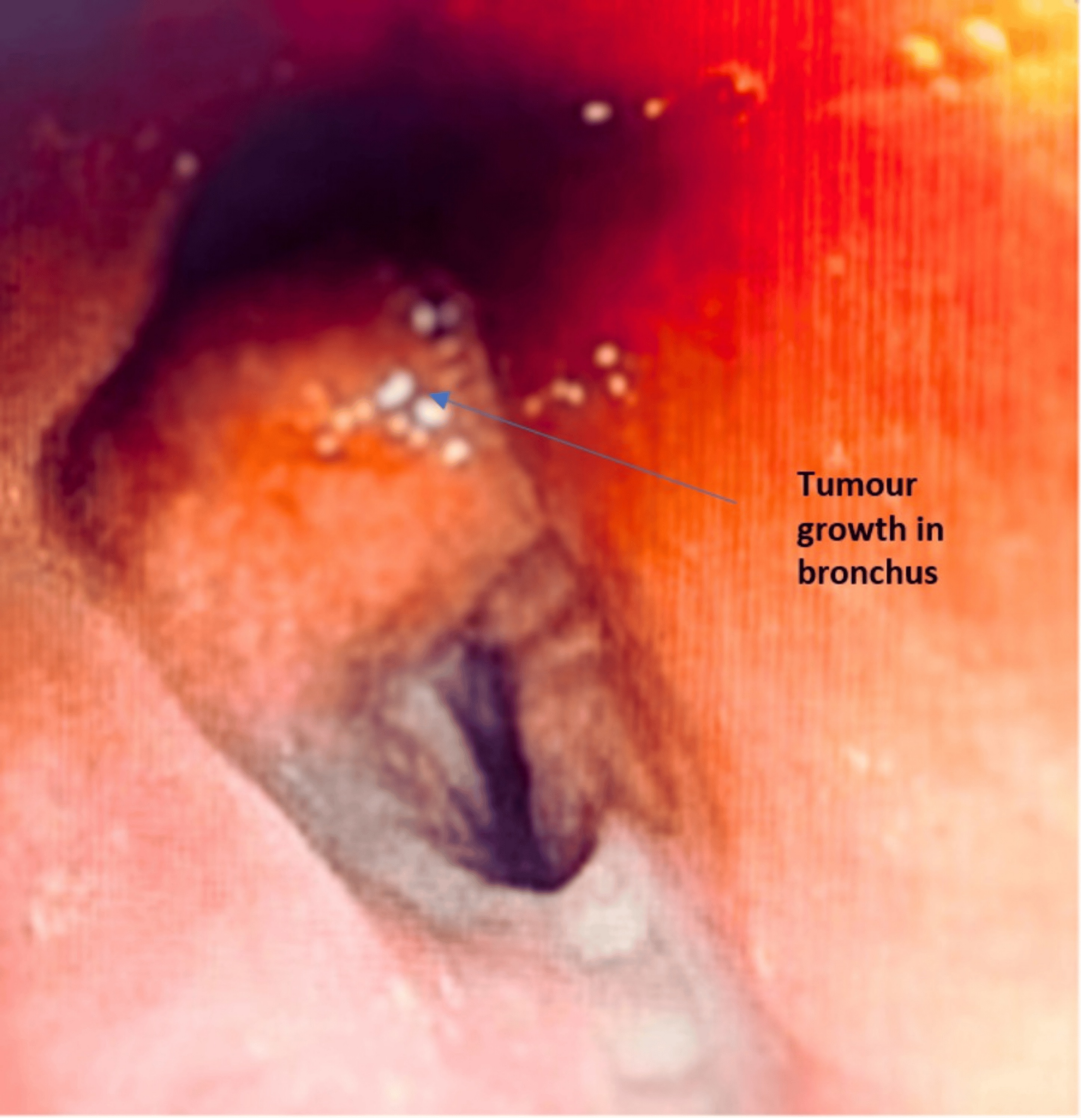
Tumor growth was observed in the bronchus of the left upper lobe

After obtaining informed consent, the patient underwent a fiberoptic bronchoscopy. During the procedure, a tumor growth or hypertrophy of the LC1 was observed in the left bronchus (Figure 3).

**Figure 4:**
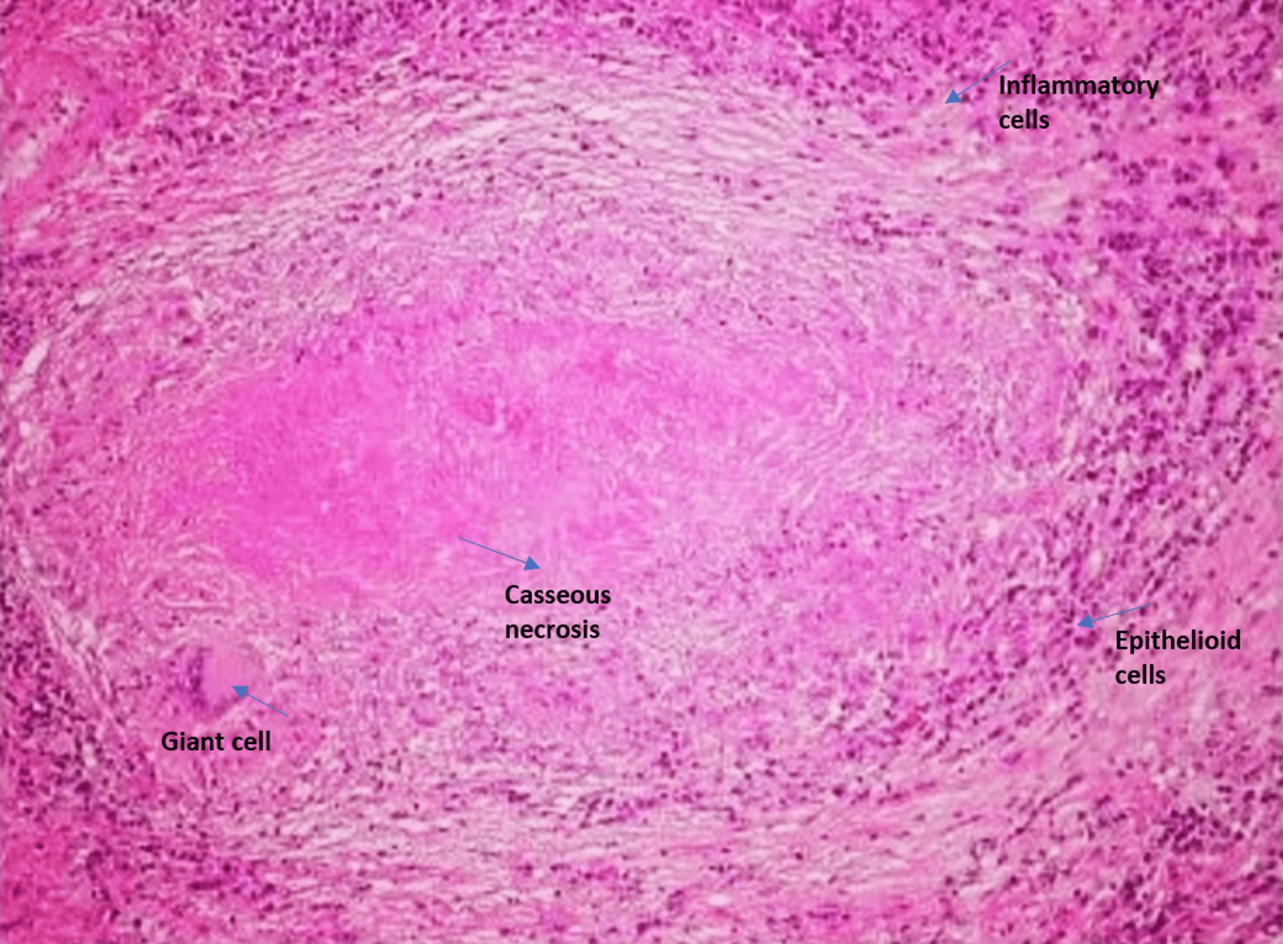
Histopathological examination (HPE) of the biopsy revealed granulomatous changes, giant cells, and epithelioid cells

A biopsy from the growth was sent for histopathological examination, revealing a granulomatous lesion (Figure 4). Bronchoalveolar lavage (BAL) samples were also sent for analysis. The BAL acid-fast bacilli (AFB) test was negative, but the BAL CBNAAT detected Mycobacterium tuberculosis (MTB), which was sensitive to rifampicin. Consequently, the patient was started on first-line anti-tuberculosis treatment (ATT).

## Discussion

Inhalation of crystalline silica dust leads to occupational pneumoconiosis, progressively promoting pulmonary fibrosis and increasing the risk of lung cancer and TB. Symptoms can manifest from a few weeks to several years after exposure, depending on the severity and duration of exposure. Occupations such as stone cutting, glassmaking, mining, and other foundry labor expose workers to significant levels of silica [6]. Silicosis is categorized based on the time of symptom onset: acute silicosis appears within a few weeks to a few years, accelerated silicosis within 10 years, and chronic silicosis more than 10 years post-exposure. Upon inhalation, crystalline silicon dioxide deposits in the bronchioles and alveoli, activating alveolar macrophages, which cause direct damage to the adjacent lung parenchyma. The crystalline form of silica is a common lung irritant found in various construction materials, while the amorphous form is less likely to cause significant clinical issues upon inhalation.

Diagnosis of silicosis is primarily through imaging techniques. Chest X-rays often show well-defined opacities in the upper lobes and posterior regions of the lungs, while CT scans reveal small, calcified nodules, hilar, and mediastinal lymphadenopathy. Pulmonary function tests may demonstrate restrictive patterns and bronchoscopy with biopsy can be utilized for further histopathological confirmation [7]. Preventative measures in workplaces include the use of personal protective equipment, dust control mechanisms, and regular health monitoring of workers exposed to silica dust. Early detection and intervention are crucial to managing silicosis and preventing its progression to more severe forms of lung disease. Public health policies and occupational safety regulations play a vital role in reducing the incidence of silicosis among workers in high-risk industries.

There is currently no effective therapy for silicosis, despite its high prevalence. Workers exposed to silica, regardless of whether they develop silicosis, have an elevated risk of TB due to their exposure to silica dust. Patients with silicosis have a significantly higher likelihood of developing TB, with risk estimates ranging from 2.8 to 39 times greater than that of healthy controls [8]. 

This increased risk is attributed to the impaired immune response caused by silica exposure. Silica particles, when inhaled, not only cause direct damage to lung tissue but also disrupt the function of alveolar macrophages, the primary immune cells responsible for engulfing and destroying pathogens, including Mycobacterium tuberculosis. The compromised function of these macrophages leads to a weakened defense against TB, making individuals with silicosis more susceptible to the infection [9].

The patient's clinical presentation was marked by a persistent cough with expectoration, intermittent breathlessness, and hemoptysis. Constitutional symptoms, including significant weight loss and poor appetite, further pointed towards a chronic, debilitating process. The patient's occupational history and lifestyle factors, such as long-term smoking and alcohol consumption, compounded his risk of developing respiratory disease [10]. Radiological silicosis developed before TB was ever suspected. A tuberculous infection of the end branches of the tracheobronchial tree is known as endobronchial TB. However, endobronchial TB is also possible in 15% of the senior population. Right upper lobe and right main bronchus involvement is prevalent in endobronchial TB. Bronchoscopy appearance categories seven subgroups of endobronchial TB: caseating, edematous-hyperamic, fibro-stenotic, tumorous, granulomatous, ulcerative, and nonspecific bronchitis [11]. Fiberoptic bronchoscopy and CT chest scans, together with microbiological and histological data, are used to confirm a diagnosis of endobronchial TB. When sputum smears come back negative for AFB, a bronchoscopy may be used to see any endobronchial abnormalities and take a biopsy. Surgical techniques including balloon dilatation and endobronchial stenting are used to treat after-effects of endobronchial TB.

Rare endobronchial tumors may manifest with a variety of symptoms and signs. Rare and displaying a wide variety of clinical manifestations, endobronchial neoplasms are characterized as tumors affecting the bronchial lumen [12]. Recently, adenocarcinoma has surpassed squamous cell carcinoma to become the most frequent kind of lung cancer. The major adult malignant endobronchial histology is squamous cell carcinomas [13,14].

Hemoptysis and obstructive pneumonia suggest intraluminal involvement of the airways by the cancer. Bronchoscopy and other imaging tests help doctors make a diagnosis. Patients with localized malignancies who decline surgical treatment may benefit from definitive radiation therapy, which may include brachytherapy. Radiation treatment and other forms of intervention might make the postponed procedure much more challenging and raise the chance of complications [15].

In our case, the patient presented with complaints of hemoptysis, and breathlessness which raised suspicion of airway obstruction; the patient also had a history of significant loss of weight, which gave an idea to consider the diagnosis as silicosis-induced endobronchial tumor or endobronchial TB. X-ray chest was done which showed bilateral diffuse infiltrates with left upper zone heterogeneous opacity and HRCT thorax was done which showed multiple nodular opacities in bilateral upper and lower lobes and soft tissue density with bronchiectasis changes in the apical and anterior segment of the left upper lobe. Bronchoscopy was done and BAL samples were sent for analysis along with a biopsy for histopathology. Histopathology reports showed granulomatous changes and BAL CBNAAT came positive; hence, the patient was started on ATT and was diagnosed with silicosis-induced TB.

## Conclusions

This case report underscores the significant diagnostic and therapeutic challenges posed by the coexistence of silicosis and endobronchial TB, a rare and complex condition. The patient's long-term exposure to silica dust, combined with a substantial smoking and alcohol history, complicated the clinical picture. The endobronchial presentation of TB, mimicking a neoplastic lesion, further added to the complexity of the diagnosis. Comprehensive diagnostic approaches, including bronchoscopy, biopsy, and advanced imaging techniques, were essential in identifying the granulomatous lesion and confirming the presence of Mycobacterium tuberculosis. The successful initiation of first-line anti-TB treatment highlights the necessity of prompt and accurate therapeutic interventions in managing such multifaceted pulmonary conditions.

This case emphasizes the critical need for heightened clinical vigilance and a multidisciplinary approach when evaluating patients with significant occupational exposures and lifestyle risk factors. It highlights the importance of integrating thorough clinical, radiological, and pathological evaluations to achieve accurate diagnoses. Additionally, this case underscores the value of bronchoscopy and biopsy in resolving diagnostic ambiguities in pulmonary diseases. Recognizing the potential for TB to mimic malignancy in patients with silicosis is vital for ensuring timely and appropriate treatment, ultimately improving patient outcomes in such complex clinical scenarios.
